# Transforming growth factor β-induced epithelial to mesenchymal transition requires the Ste20-like kinase SLK independently of its catalytic activity

**DOI:** 10.18632/oncotarget.21928

**Published:** 2017-10-19

**Authors:** Jillian Conway, Khalid N. Al-Zahrani, Benjamin R. Pryce, John Abou-Hamad, Luc A. Sabourin

**Affiliations:** ^1^ University of Ottawa, Department of Cellular and Molecular Medicine, Ottawa, Ontario, K1H8M5, Canada; ^2^ Ottawa Hospital Research Institute, Cancer Therapeutics, Ottawa, Ontario, K1H8L6, Canada

**Keywords:** SLK, Stk2, EMT, TGFβ, invasion

## Abstract

Invasion can be stimulated *in vitro* using the soluble ligand transforming growth factor-β (TGFβ) to induce a process called epithelial-to-mesenchymal transition (EMT) characterized by cell-cell junction breakdown and an invasive phenotype. We have previously demonstrated a role for Ste20-like kinase SLK cell migration and invasion. Here we show that SLK depletion in NMuMG mammary epithelial cells significantly impairs their TGFβ-induced migration and invasion. Immunofluorescence studies show that a fraction of SLK localizes to E-cadherin-positive adherens junction and that SLK impairs the breakdown of cell-cell contacts. We find that SLK-depleted cultures express significantly lower levels of vimentin protein as well as Snai1 and E-cadherin mRNA levels following TGF-β treatment. Surprisingly, our data show that SLK depletion does not affect the activation and nuclear translocation of Smad3. Furthermore, we show that expression of a dominant negative kinase does not impair tight junction breakdown and rescues Snai1 mRNA expression levels. Together these data suggest that SLK plays a novel role in TGFβ-induced EMT, independent of Smads, in a kinase activity-independent manner.

## INTRODUCTION

Epithelial-to-mesenchymal transition (EMT) is essential for both normal growth processes, including wound healing, and tissue regeneration [1, 2]. EMT also plays an important role in embryogenesis and tissue morphogenesis [3]. During EMT, cells acquire a more motile and invasive phenotype through the breakdown of intercellular contacts such as desmosomes, adherens junctions, and tight junctions [4]. These cell-cell contacts consist of a variety of proteins that contribute to the maintenance of the epithelial structure and polarity [5]. In normal epithelial tissues, intercellular contacts function as an antagonist of invasion and metastasis by maintaining cell quiescence and the assembly of epithelial sheets [6, 7]. The disintegration and reorganization of this epithelial phenotype leads to a more motile and invasive cell type found in mesenchymal cells and invasive tumors. The phenotypic change from epithelial to mesenchymal cells is characterized by distinct changes in cell morphology: a decreased expression of adherens and tight junction markers such as E-cadherin and ZO-1, respectively; an increased expression of mesenchymal markers (i.e. fibronectin and N-cadherin) and a re-organization of actin fibers in the cytoskeleton [8].

EMT can be stimulated by many factors including Epidermal growth factor (EGF), Hepatocyte growth factor (HGF), Fibroblast growth factor (FGF), bone morphogenetic proteins (BMP), Notch, Wnt, TNF-α and multiple cytokines [9, 10]. EMT can also be induced by transforming growth factor beta-1 (TGFβ-1) both *in vivo* and *in vitro* [8]. Activation of the EMT process by TGFβ induces the phosphorylation and activation of the Smad proteins [11–15]. Their translocation to the nucleus activates a genetic program associated with the morphological changes that occur during EMT [16–18]. These include the breakdown and downregulation of E-Cadherin as well as the upregulation of transcription factors of the Snail, Zeb and Twist families [19]. The mesenchymal phenotype is also accompanied by the expression of Fibronectin, Vimentin, N-Cadherin and the matrix metalloproteinases MMP2/9 [20].

Cancer metastasis is the cause of 90% of human cancer deaths, making this an important field of study in research [7, 21]. Metastasis of a primary tumor, leading to the colonization of distant organs and the establishment of secondary tumors, relies on the process of EMT [8]. For cancer cells to successfully metastasize, they must first breach the basement membrane, intravasate into the lumen of blood or lymph vessels, evade the body's immune cells in circulation, extravasate into a distant tissue, and re-colonize at a distant site [22]. The reverse process of EMT, mesenchymal-to-epithelial transition (MET), is required for generating tumor growths at a secondary site [23].

Cell migration is an important regulatory process involved in embryogenesis, the inflammatory response, and tissue repair and regeneration throughout the normal cell life cycle [24, 25]. The later stages of EMT are characterized by increased cell migration and cytoskeletal reorganization. However, the inappropriate activation or dysregulation of this process is an important step in the process of EMT in cancer cells [26, 27]. We have previously demonstrated that the Ste20-like kinase SLK [28, 29] plays a role in a variety of cellular processes (reviewed in [30]), including apoptosis, cell cycle progression [31], cell migration [32, 33], HER2 signaling [34], muscle function [35, 36] and embryonic development [37]. The knock down of SLK inhibits scratch-induced cell migration [33] and focal adhesion turnover [38]. As SLK plays a role in cytoskeletal remodelling, cell migration and heregulin-induced invasion of breast cancer cells [34], we have tested the hypothesis that SLK is required for TGFβ-mediated EMT and the downstream cytoskeletal remodelling necessary to confer the invasive phenotype.

Our data show that SLK depletion impairs the cytoskeletal change associated with TGFβ-induced EMT without blocking Smad activation. SLK knock down impairs E-Cadherin downregulation as well as Snail, Fibronectin and vimentin induction. This is also accompanied by a decrease in TGFβ-induced invasiveness and motility. Surprisingly, SLK activity is not modulated by TGFβ treatment and the expression of kinase inactive SLK does not impair the expression of Snail, suggesting that SLK plays a kinase activity-independent scaffolding function during TGFβ-mediated EMT.

## RESULTS

### SLK knock down impairs TGFβ-driven motility and invasion

**Figure 1:**
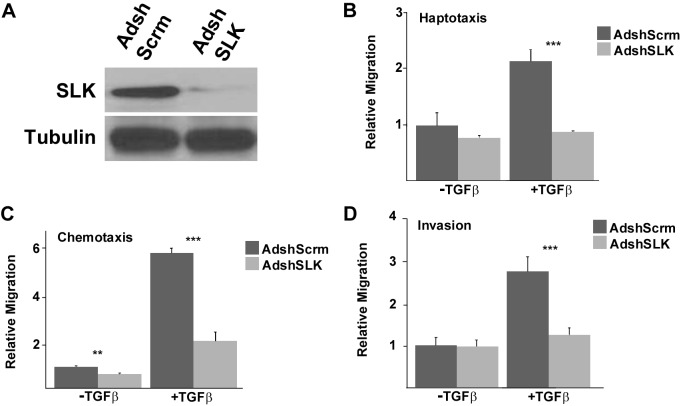
SLK depletion inhibits TGF β-induced motility and invasion **(A)** NMuMG cells were first infected with AdshScrambled or AdshSLK adenovirus for 48 hours prior to TGFβ1 treatment and SLK expression was assessed by Western blot. **(B)** Following TGFβ treatment (2ng.ml for 48h), 50,000 cells were seeded into fibronectin-coated Boyden chambers in 0.2% serum and allowed to migrate for 24 hours. Cells that migrated to the fibronectin side were enumerated. **(C)** The chemotactic assay was performed as in (A) with the addition of 2 ng/ml TGFβ in the bottom chamber. **(D)** The invasion assay was performed as in (A) onto matrigel-coated chambers. All assays were performed in triplicate and were counted relative to the untreated scramble control. ^*^p<0.05, ^**^p<0.01, ^***^p<0.001

During EMT, cells transition from an apical-basal polarity to a front-back polarity [39]. Cells that undergo EMT present with a more invasive phenotype [2, 7]. We have previously shown that SLK is required for cell motility and heregulin-driven chemotaxis and invasion [33, 34]. Because of our interest the response of mammary epithelium and breast cancer cells to pro-invasive signals, we investigated whether SLK knockdown would also impair the increased motility phenotype associated with TGFβ-induced EMT. To test this, we have used NMuMG mammary epithelial cells. NMuMG cells were originally isolated as an adherent epithelial cell line from benign murine cystadenomas that undergo EMT in the presence of TGFβ [15, 40, 41]. NMuMG cells were infected with GFP-tagged Ad-scrambled or AdshSLK prior to TGFβ treatment. A marked downregulation (≥ 90%) was achieved at low MOI and persisted for up to 9 days (Figure 1A and Supplementary Figure 1). The infected cells were then treated with TGFβ for 48 hours to induce EMT and plated in a Boyden chamber to assess migration and invasion. In the absence of SLK, NMuMG cells showed a three-fold decrease in their haptotactic capacity towards a fibronectin-coated membrane (Figure 1B). Similarly, in a chemotaxis assay, the migration towards a gradient of TGFβ was decreased two-fold in the absence of SLK (Figure 1C). SLK knock down was previously shown to inhibit heregulin-driven invasion in breast cancer cells [34]. Therefore, the role of SLK in TGFβ-induced invasion was also analyzed using matrigel-coated chambers. As observed previously for heregulin, SLK knockdown showed a 2.5-fold reduction in TGFβ-driven invasion through matrigel-coated substrates (Figure 1D). These data strongly suggest that SLK is required for TGFβ-driven motility and invasion in mammary epithelial cells.

**Figure 2:**
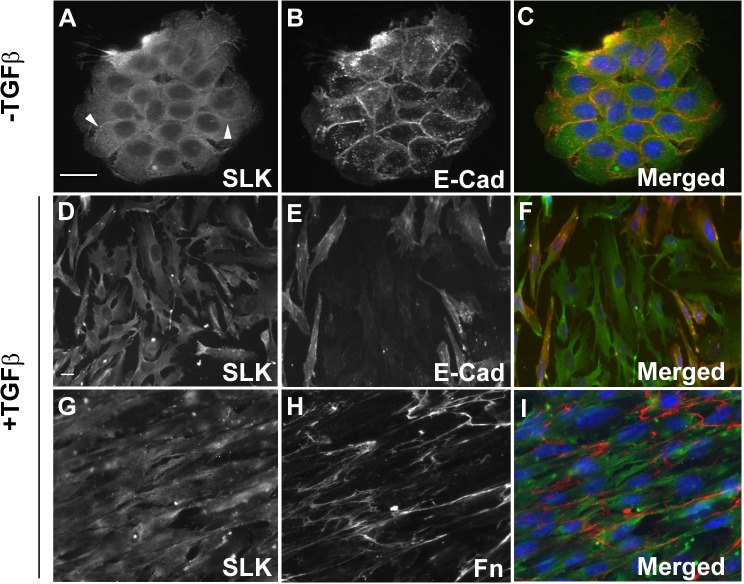
Localization of SLK in murine mammary epithelial cell NMuMG cells were subjected to immunofluorescence studies before and after TGFβ1 stimulation for 48 hours (2ng/ml). Double immunostaining was performed for SLK **(A&D)** and E-cadherin **(B&E)** or SLK **(G)** and Fibronectin (**H**; Fn). Merged panels **(C, F, I)** show SLK (green) and E-Cadherin (red) or Fibronectin (red). Nuclei were visualized with DAPI (blue). The E-Cad/SLK overlapping signal accounted for approximately 15% of total SLK.

Because of its role in focal adhesion turnover [33], one possibility is that SLK depletion inhibits the cell motility response induced by TGFβ. Alternatively, SLK may be required for the initial cytoskeletal changes induced by TGFβ stimulation. Prior to investigating its role in EMT, the cellular distribution of SLK was examined in unstimulated and exponentially growing NMuMG mammary epithelial cells. Co-immunostaining with both epithelial and mesenchymal markers showed a diffuse cytosolic SLK pattern with increased reactivity at the cell-cell junctions, co-localizing with E-cadherin, an epithelial cell marker of adherens junctions (about 15 ± 3% co-localization; Figure 2A–2C). Upon TGFβ1 treatment, SLK was found to be cytosolic but predominantly re-distributed to ruffles, lamellipodia and cytosolic extensions of migrating cells (Figure 2D&2G). This has been previously observed in migrating fibroblasts [33]. Concomitantly, we observed a re-distribution in E-cadherin staining (Figure 2E) whereas fibronectin, a mesenchymal marker, was markedly upregulated (Figure 2H). Together these results suggest that, in epithelial cells, a fraction of SLK is associated with the adherens junction and is redistributed upon TGFβ-induced EMT.

**Figure 3:**
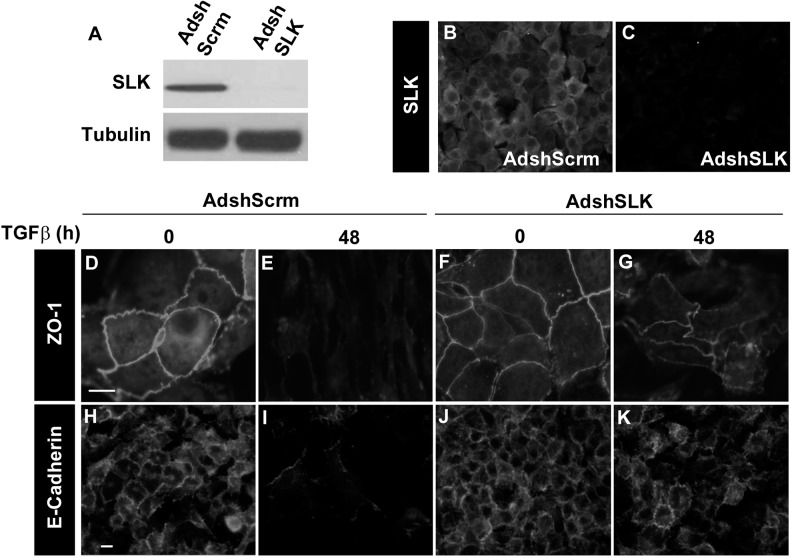
SLK depletion inhibits EMT-associated cytoskeletal changes NMuMG cells were infected with either Adsh-Scrambled or AdshSLK for 48 hours. SLK expression was monitored by Western blot **(A)** and Immunostaining **(B&C)**. The cells were then stimulated with TGFβ1 for an additional 48 hours and stained using anti-ZO-1 **(D-G)** or E-cadherin **(H-K)**. Scale bar=10μm. SLK knock down was found to block the breakdown of ZO-1 and E-cadherin-positive junctions.

TGFβ stimulation and activation of EMT signaling result in dramatic morphological changes, including the breakdown of adherens junctions and tight junctions. Adherens junctions are characterized by E-cadherin-positive structures formed through homophilic interactions between E-cadherin molecules from adjacent cells. Tight junctions (TJ) are positive for Zonula occludens-1 (ZO-1) peripheral membrane protein that scaffolds and anchors the tight junctions to the actin cytoskeleton [39]. The EMT process is accompanied by the downregulation of the epithelial cell markers E-cadherin and ZO-1 [39]. As a fraction of SLK localizes with E-cadherin at the adherens junctions, we assessed the effect of SLK knock down on the cytoskeletal changes associated with TGFβ-induced EMT in NMuMG cells. Prior to TGFβ stimulation, the cells were infected with a scrambled control or shSLK adenovirus to knockdown SLK expression (Figure 3A–3C). At 48 hours following infection, the cultures were treated with 2ng/mL of TGFβ1 for two days to induce EMT. The cells were then immunostained for E-cadherin and ZO-1 to assess the status of the adherens and tight junction, respectively. A 48 hour TGFβ treatment induced the complete disassembly of ZO-1 positive junctions in scrambled-infected cells (Figure 3E) whereas downregulation of SLK resulted in a marked inhibition in the breakdown of ZO-1-positive tight junctions (Figure 3G). Similarly, a rapid breakdown of E-cadherin-positive adherens junctions was observed in control cultures (Figure 3I) that was inhibited by SLK knock down with the maintenance of epithelial morphology (Figure 3K). Together, these data suggest that SLK is required for the morphological changes that occur during TGFβ-induced EMT.

### SLK depletion does not impair Smad activation

The canonical TGFβ signaling pathway inducing EMT involves multiple dimeric proteins called Smads that participate in a signaling cascade that ultimately results in both cytoskeletal and genetic changes in the cell [42]. Smads 2 and 3 get phosphorylated by the TGFβR complex and form a trimer with the Smad 4 protein. This complex then translocates into the nucleus and binds to DNA to exert its effects through transcriptional activation of mesenchymal markers and transcriptional repression of epithelial markers [11]. To further investigate the role of SLK downstream of TGFβ signalling, the activation status of the canonical pathway was assessed in the absence of SLK.

**Figure 4:**
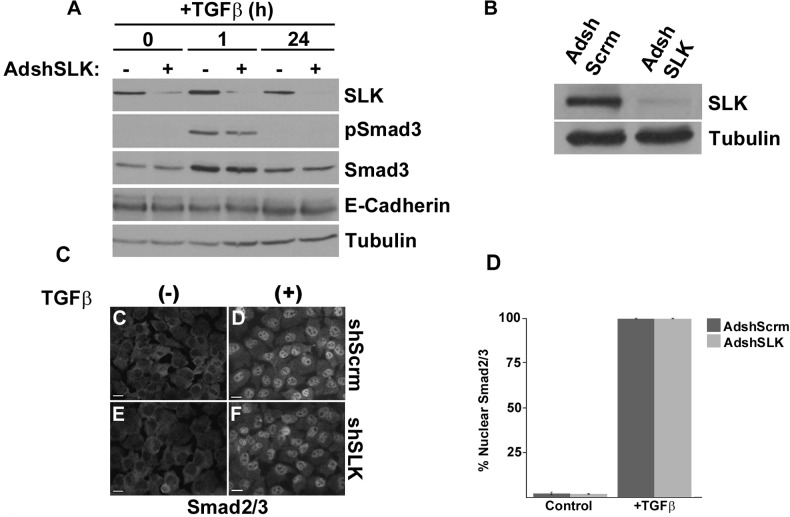
SLK depletion does not affect R-Smad activation **(A)** NMuMG cells were infected with AdshScrambled or AdshSLK for 48 hours prior to treatment with 2ng/mL of TGFβ1 for 1 or 24 hours. Western analysis was used to assess the levels of SLK, phospho-Smad3 (pSmad3), total Smad3 and E-Cadherin. NMuMG cells infected with AdshScrambled or AdshSLK **(B)** were stimulated with TGFβ1 for one hour (+) and subjected to immunofluorescence using anti-Smad2/3 antibodies **(C)**. Scale bar=10μm. **(D)** Quantification of Smad2/3 nuclear localization expression relative to the total number of nuclei obtained by DAPI staining. Error bars represent the standard error.

We first explored the effect of SLK knockdown on receptor-Smad phosphorylation and protein stability. We first knocked down SLK expression in NMuMG and treated the cells with 2ng/mL of TGFβ1 for 0, 1 or 24 hours to stimulate the EMT process. Surprisingly, Western bot analysis showed that the levels of phospho Smad3 or total Smad3 were unaffected by SLK depletion (Figure 4A). This supports the notion that SLK does not regulate EMT upstream of R-Smad activation.

The Smad complex must translocate into the nucleus to drive gene transcription associated with the EMT response [43–45]. One possibility is that SLK knock down impairs the translocation of the Smad complex to the nucleus, preventing the downregulation of E-cadherin. To asses this, we performed immunofluorescence and cellular fractionation studies. SLK was knocked down in NMuMG cells for 48 hours and the cells were treated with 2ng/mL of TGFβ1. Following this, the nuclear translocation of Smad3 was assessed. Immunostaining for phospho-Smad3 showed no quantitative difference in the levels of nuclear Smad3 between AdshSLK and Ad-scrambled infected cells, suggesting that SLK depletion does not impair the shuttling of the Smad/2/3/4 transcriptional complex (Figure 4B–4G). Supporting this, cellular fractionation studies revealed no significant differences in the levels of nuclear Smad3 between AdshScramble and AdshSLK-treated cells (Supplementary Figure 2). These results suggest that SLK regulates TGFβ-induced EMT independently of R-Smad activation and transclocation.

### SLK is required for the induction of a subset of TGFβ-regulated genes during EMT

**Figure 5:**
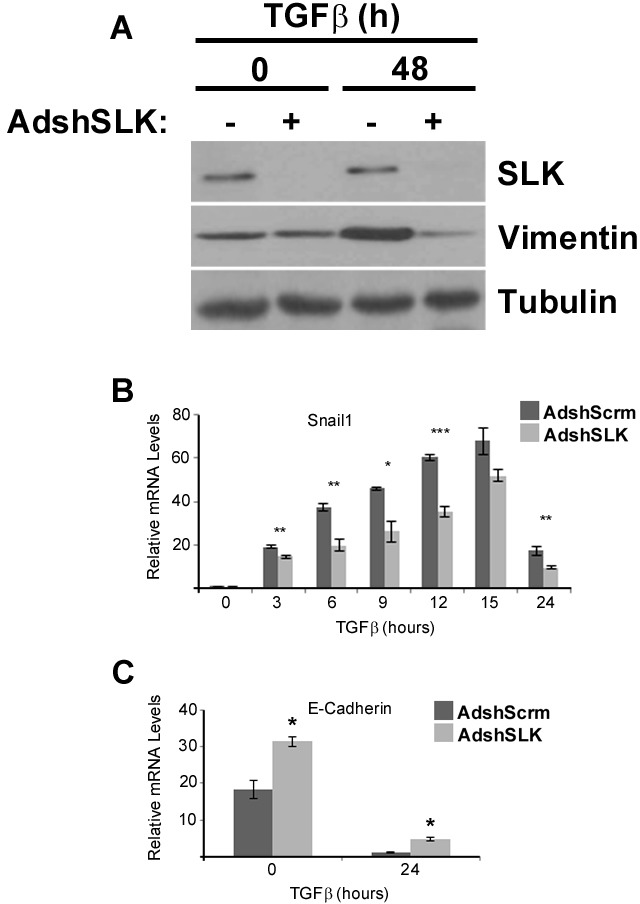
SLK knockdown significantly inhibits Snai1 and vimentin expression following TGFβ1 treatment **(A)** NMuMG cells were infected with either AdshScrambled or AdshSLK for 48 hours. The cultures were stimulated with TGFβ and with and surveyed for SLK and vimentin expression. (B) Total RNA was extracted from identical cultures and Snai1 **(B)** and E-Cadherin **(C)** mRNA levels were monitored by Q-PCR. Normalization was performed against GAPDH mRNA levels. Each experiment was run in triplicate with three biological replicates.^*^p<0.05, ^**^p<0.01, ^***^p<0.001

Following TGFβ1 treatment in NMuMG cells, epithelial markers such as E-cadherin become downregulated and mesenchymal markers like vimentin are induced [18]. Western analysis showed that SLK knock down suppressed vimentin expression two days following TGFβ treatment (Figure 5A), suggesting a delay in EMT. To gain further insights into the role of SLK in TGFβ-mediated EMT we investigated the genetic response downstream of TGFβ treatment. We performed qPCR to evaluate the mRNA levels of target genes shown to be modulated following TGFβ stimulation. Cultures were infected with control and shSLK viruses and then treated with TGFβ1. Total RNA was extracted every 3 hours for 15 hours and subjected to qPCR analysis. Interestingly, our results show that in the absence of SLK, Snai1 expression [46] was significantly reduced throughout the time course compared to the scrambled control sample (Figure 5B). However, Snai1 protein levels or distribution were not altered during the time course (Supplementary Figure 3), suggesting that the expression of downstream targets is not affected. Snai2 (Slug), Twist2 or MMP9 gene expression did not change significantly in the absence of SLK (Supplementary Figure 4). Analysis for E-cadherin expression showed that, although it was down regulated, its expression was significantly higher following a 24-hourTGFβ treatment in the absence of SLK (Figure 5C), suggesting that its complete downregulation is also impaired in the absence of SLK. Notably, prior to TGFβ treatment, the E-cadherin levels in the SLK-depleted cells were higher than control. Together, these data suggest that SLK downregulation interferes with the activation and repression of a subset of EMT target genes, likely resulting in an impaired EMT response.

### SLK regulates EMT in a kinase activity-independent manner

**Figure 6:**
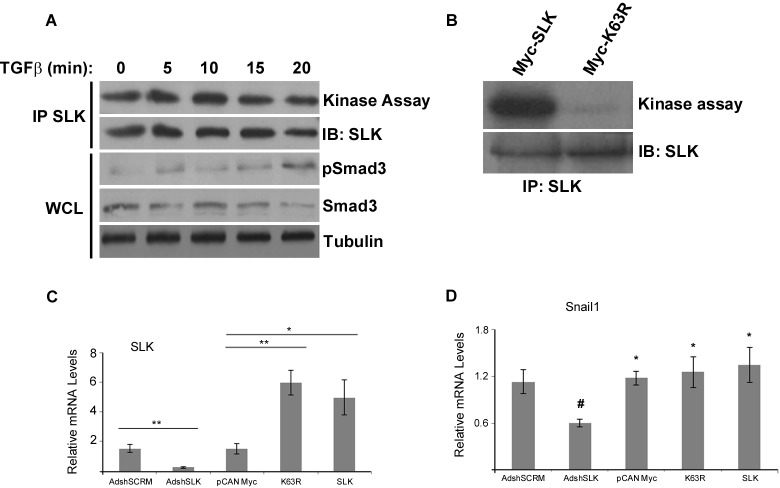
SLK regulates EMT independently of its kinase activity **(A)** NMuMG cells were treated with 2ng/mL of TGFβ1 for various times and SLK was immunoprecipitated and subjected to *in vitro* kinase assays. IP= immunoprecipitate, IB= immunoblot, WCL= whole cell lysate. **(B)** NMuMG cells were transfected with a wildtype or dominant negative (K63R) SLK construct and total SLK was immunoprecipitated and assayed for kinase activity. IB= immunoblot. Total RNA was also extracted from the transfected cultures following TGFβ stimulation (2ng/ml for 9 hours) and assayed for SLK **(C)** or Snai1 **(D)** expression. mRNA levels were normalized to GAPDH and directly compared to AdshSLK-infected cultures. Each experiment was run in triplicate with three biological replicates. Error bars represent the standard error. ^*^p<0.05, ^**^p<0.01

As SLK depletion impairs cell-cell contact breakdown and TGFβ-driven motility, we tested whether TGFβ stimulation induces its kinase activity. NMuMG cells were serum-starved and treated with TGFβ for various times. The cells were harvested, lysed and assessed for SLK kinase activity using *in vitro* kinase assays. Whereas pSmad3 was induced within 20 minutes of TGFβ stimulation, kinase assays showed that SLK activity did not change over the time course following stimulation with TGFβ1 (Figure 6A). Similarly, longer time courses showed no activation of SLK (not shown), suggesting that TGFβ stimulation does not upregulate SLK activity.

As TGFβ stimulation does not modulate SLK activity, one likely possibility is that SLK affects EMT signaling through a scaffolding function. Activation of SLK occurs through homodimerization in a *trans* orientation, resulting in autophosphorylation and activation [47–50]. A point mutation at lysine 63 to an arginine (hereby referred to as K63R) inactivates the full-length kinase and acts as a dominant negative protein, allowing dimerization with endogenous SLK to inactivate the entire complex [29]. Therefore, to test the requirement for SLK activity during EMT, a kinase-dead version of SLK (K63R) was expressed into NMuMG cells at high levels. Immunoprecipitation and kinase assays of transfected NMuMG cells show that expression of K63R markedly reduces the total SLK activity as assessed by autophosphorylation (Figure 6B). Expression of the K63R mutant resulted in a 6-fold overexpression of the mutant kinase mRNA (Figure 6C). Surprisingly, in contrast to a 50% reduction in Snai1 expression in AdshSLK-infected cultures, K63R expression had no effect on Snai1 mRNA levels upon TGFβ treatment (Figure 6D). Supporting this, expression of K63R also had no effect on the breakdown of ZO-1-positive tight junctions upon TGFβ stimulation (Supplementary Figure 5). These data strongly suggest that SLK plays a kinase activity-independent role in TGFβ-induced EMT. The observation that overexpression of wildtype SLK did not increase Snai1 mRNA levels also suggest that it plays a scaffolding role in a complex containing limiting components.

## DISCUSSION

EMT is characterized by a loss in epithelial cell markers (e.g. E-Cadherin and ZO-1) and an increase in the expression of mesenchymal markers [3]. It has been previously demonstrated that SLK localizes to the cell periphery, specifically at the leading edge of migrating fibroblasts cells [33]. Furthermore, SLK depletion results in impaired cell migration and focal adhesion turnover [33, 38]. Here we show that SLK depletion inhibits TGFβ-induced migration and invasion through matrigel coated substrates (Figure 1). One likely explanation is that the observed defect in migration results from a delay in cytoskeletal remodelling and focal adhesion turnover as previously demonstrated [33, 38].

Immunostaining of NMuMG mammary epithelial cells show that a fraction of SLK co-localizes with E-cadherin at the adherens junction which is redistributed to the cell periphery upon TGFβ stimulation (Figure 2). One possibility is that SLK might play a role in adherens junctions stability or organization. Alternatively, it might be sequestered at adherens junction in non-motile cells.

Using a short hairpin adenovirus that reduce SLK expression by >90%, we have observed that SLK depletion inhibited the breakdown of E-cadherin and ZO-1 positive adherens and tight junction, respectively without affecting Smad3 activation or nuclear translocation (Figures 3 and Figure 4). These results suggest that SLK is required for the cytoskeletal changes induced by TGFβ treatment in a Smad-independent manner. Our results show that E-cadherin expression was not completely downregulated following SLK depletion. Interestingly, Snai1 expression was inhibited by about 50% in the absence of SLK (Figure 5) whereas Slug, Twist2 and MMP9 gene activation remained unaffected; suggesting that SLK depletion preferentially affects specific genetic responses. Interestingly, Snai1 protein levels and distribution remained unchanged upon SLK knock down, suggesting that Snai1 activity might not be affected (Supplementary Figure 3). However, vimentin expression was not upregulated in the absence of SLK suggesting the cells cannot fully transition into a mesenchymal phenotype [18] and that SLK depletion only affects a subset of EMT-responsive targets. Combined with the role of SLK in focal adhesion turnover, these observations suggest that SLK is critical for TGFβ-induced cytoskeletal reorganization and full EMT. One possibility is that the failure to breakdown cell-cell contacts in the absence of SLK impairs the activation of specific transcriptional activation mechanisms or nuclear translocation of co-factors. During EMT, a large number of cytoskeletal changes have been shown to occur [51] such as alterations in microtubule dynamics and expression of cytoskeletal-associated genes. Cytoskeletal changes such as adherens junction breakdown, have been shown to regulate downstream transcription factors such as YAP (reviewed in [52]). In addition, the activation of numerous non-canonical Smad-independent pathways, including Ras/Erk/p38 MAPK, PI3K/Akt and Rho-like GTPase pathways (reviewed in [18, 53]). These pathways are involved in key transcriptional events that are necessary for the cytoskeletal and genetic changes that occur during the EMT signaling pathway. It is then likely that SLK is acting downstream of Smad-independent systems to regulate cytoskeletal-dependent transcriptional control leading to gene activation. To our knowledge this is the first evidence that SLK plays a role in a transcriptional response.

It has been well documented that Snai1 is critical for silencing E-Cadherin gene expression by binding directly to the E-box [20]. We have observed that shSLK-treated cells express higher E-Cadherin levels prior to TGFβ treatment and do not completely downregulate E-Cadherin upon TGFβ treatment (Figure 5). As E-Cadherin expression is regulated by multiple transcription factors [20], it is possible that the failure to fully downregulate E-cadherin is due to the preferential inactivation of those factors.

Although SLK depletion inhibited the cytoskeletal changes associated with the process of EMT, our results demonstrate that SLK activity was not modulated following TGFβ stimulation (Figure 6), suggesting that SLK regulates EMT independently of its kinase activity. Supporting this, expression of a dominant negative SLK [29] previously shown to inhibit fibroblast cell migration [33], cell proliferation [31] and myoblast fusion [54] had no effect on Snai1 expression or tight junction breakdown upon TGFβ stimulation (Figure 6 and Supplementary Figure 5). This suggests a mechanism whereby scaffolding by SLK is required for EMT, independently of its kinase activity. One possibility is that SLK scaffolds protein interactions required for cell-cell-junction breakdown or non-canonical signaling systems required for EMT target gene activation such as Snai1 and vimentin.

Overall, our data show that SLK is required for TGFβ-induced cytoskeletal changes and the full genetic program associated with EMT. Interestingly, SLK does not mediate those changes through Smad protein inhibition or its kinase activity. Together, these results suggest a novel role for SLK in TGFβ-induced epithelial-to-mesenchymal transition and provide novel insights into kinase activity independent functions for this kinase. The isolation of SLK binding proteins and signal transducers will allow the identification and potential inhibition of these pathways to suppress EMT and cancer progression.

## MATERIALS AND METHODS

### Cell culture

NMuMG cells (ATCC, Manassas, Virginia, United States) were maintained at 37°C and 5% CO_2_ in DMEM (Dulbecco's Modification of Eagle's Medium), supplemented with 10% fetal bovine serum (FBS) (Invitrogen), 10μg/mL insulin (Roche), 2mM L-glutamine (Invitrogen), and 200 U mL^-1^ penicillin/streptomycin (Invitrogen). All experiments were performed in medium as described above but without the added insulin. The cells were serum-starved overnight prior to treatment with TGFβ1. For transfection, NMuMGs were seeded at 3 × 10^6^ cells on a 10cm plate and grown overnight to 70-90% confluency. The cells were transfected with Lipofectamine 3000™ Reagent in serum-free media, adjusted for a 10 cm plate according to the manufacturer's instructions. The transfection mix was then added to the adherent cells and topped up with 10% FBS DMEM after 3 hours. The cells were harvested 48 hours following transfection. TGFβ1 (Sigma-Aldrich) was added to the cells at a concentration of 2ng/mL in serum-free media (1X DMEM, Corning, USA) in all experiments.

### Adenovirus infection

To effectively knockdown SLK expression, GFP-tagged short hairpin adenovirus against murine SLK (AdshSLK) and a corresponding scramble control were used to infect NMuMG cells at an MOI of 20. The targeting sequence for the shSLK adenovirus (5′-GGTTGAGATTGACATATTA-3′) was previously demonstrated to efficiently knock down SLK in murine fibroblasts [33]. The cells were plated on a 10cm dish at 7.5 × 10^5^ cells/plate the day before adenovirus infection. On the day of the infection, the cells were first washed with 1X PBS (HyClone), and then re-suspended in serum-free DMEM. The purified viruses were added to the suspended cells in serum-free media and then plated at 37°C. The plates were gently agitated every 15 minutes for 90 minutes. Finally, the cells were re-fed with complete growth medium without insulin and incubated for 48 hours before harvesting.

### Protein extraction and Western blotting

For protein extraction, cells were washed in 5 mL of 1X PBS, collected using a cell scraper into 0.5 mL 1X PBS, centrifuged and then lysed using RIPA buffer (1M NaF, 1M β- glycerophosphate, 1M DTT, 0.2M NaVO_3_, 0.1M PMSF, 1mg/mL leupeptin, 1mg/mL pepstatin, 1mg/mL aprotinin, 100μM benzamide, 1M Tris, 0.05% SDS, 1% Triton X-100, 1% Igepal CA-630, 50 mM Tris-HCl, pH 7.5, 150 mM NaCl, 2 mM EDTA, pH 8.0, and 12 mM Na-Deoxycholate). Lysates were spun at 14,000rpm for 10 minutes to pellet the cell debris. The cleared lysates were then assayed for protein concentration using a Bradford Lowry Reagent (Bio-Rad, Mississauga, Ontario, Canada). For western blotting, 40μg of protein was electrophoresed on an 8% polyacrylamide gel and transferred onto a PVDF (polyvinylidene difluoride) membrane (Thermo Fisher Scientific, USA). Membranes were blocked in 5% BSA (bovine serum albumin) (Sigma-Aldrich, Oakville, Ontario, Canada) in 1X TBST (50 mM Tris, pH 7.4, 150 mM NaCl, 0.05% Tween 20) for one hour and primary antibodies were added in 5% BSA in 1X TBST for one hour at room temperature. Membranes were washed in TBST and reactive proteins were detected using horseradish peroxidase-coupled secondary antibodies (Bio-Rad, USA) and Western Lightning Plus enhanced chemiluminescence (Perkin-Elmer, USA). The following primary antibodies were used: anti-ZO-1 (Invitrogen, Camarillo, California), anti-E-cadherin (BD Transduction Laboratories, Canada), anti-SLK [33], anti-α-tubulin (Sigma-Aldrich, Oakville, Ontario, Canada), anti-Smad3 (Cell Signalling, USA), anti-phospho-Smad3 (Cell Signalling, USA), anti-Smad2/3 (Cell Signalling, USA), anti-LaminA/C (Cell Signaling, USA), anti-GAPDH (Abcam, USA), and anti-vimentin (Abcam, USA).

### Immunofluorescence

Cells were plated onto cover slips and grown overnight at 37°C in 5% CO_2_ in growth medium. Prior to staining, the cover slips were washed with PBS, fixed in 4% PFA for 10 minutes and permeabilized with 0.1% Triton X in PBS for five minutes. The coverslips were then blocked in 5% goat serum (Sigma-Aldrich, USA) in PBS and then incubated with primary antibody at room temperature. Antigens were detected using fluorescently labeled secondary antibodies. To stain for F-actin, a fluorescent conjugate of phalloidin was used (ThermoFisher Scientific, USA). Cover slips were then mounted onto microscope slides using a drop of ProLong Gold antifade reagent with DAPI (4,6-diamidino-2-phenylindole) (Invitrogen, USA). Slides were visualized using a Zeiss AxioCam fluorescence microscope.

### Transwell migration and invasion assays

Prior to plating into migration chambers, cells were treated with either shSLK or a sh-scrambled adenovirus for 48 hours, as described above. The cells were treated with 2ng/mL of TGF-β for 48 hours and 5.0 × 10^4^ cells were plated into the top part of each fibronectin-coated chamber (8 μm pores; Fisher Scientific, USA). Haptotaxis assays were run in 0.2% FBS DMEM media. Chemotactic assays were run with the bottom chamber containing TGF-β. The migration chambers were placed at 37°C at 5% CO_2_ for six hours. Each well was washed and fixed in 4% PFA for ten minutes. The washed membranes were removed and placed cell-side up onto a microscope slide. Each membrane was covered in ProLong Gold antifade reagent with DAPI and the migrated cells were enumerated using fluorescence microscopy. The assays were done in triplicate wells the cells were counted from five random fields per membrane. Invasion assays were performed as above but for 24 hours using Matrigel-coated invasion chambers (BD Biosciences, Canada).

### Immunoprecipitation and kinase assays

NMuMG cells were plated at a concentration of 3.0 × 10^6^ cells/ 10 cm plate and incubated for 24 hours. The cells were then serum-starved overnight and treated with 2ng/mL of TGFβ in serum-free media for various times. Cells lysates were collected as described above and 400μg of protein was subjected to immunoprecipitation with anti-SLK antibodies and 20μL of Protein A agarose (GE Healthcare, USA) as previously described [33]. The precipitates were washed in NETN (20mM Tris pH 8, 1mM EDTA pH 8, 200mM NaCl, 0.5% NP-40) and once in SLK kinase buffer (0.02M Tris pH 7.4, 0.001M NaF, 0.01M β-Glycerophosphate, 0.001M DTT, 0.015M MgCl_2_, 250μM NaVO_3_). *In vitro* kinase assays were initiated by the addition of ^32^P-γATP (5 μCi, Perkin Elmer, USA) in 20μL of SLK kinase buffer. The reactions were incubated at 30°C for 30 minutes and terminated with the addition of 7μL of 4X SDS sample buffer (0.2M Tris pH 7.4, 0.4M DTT, 8% SDS, 4mL glycerol, 6mM bromophenol blue). The samples were boiled and loaded onto 8% polyacrylamide gels, transferred onto a PVDF membrane and exposed to X-ray film.

### RNA extraction and quantitative PCR analysis

NMuMG cells were plated at 3 × 10^6^cells per 10 cm plate and grown for 24 hours. The cells were then serum-starved overnight and treated with 2ng/mL of TGFβ1 (Sigma-Aldrich, USA) for the indicated time points. Total RNA was extracted using Trizol (Ambion, Life Technologies, USA) as per the manufacturer's protocol. The final RNA was re-suspended in RNAse-free water for use in cDNA synthesis. To ensure no DNA contamination, the samples were run through the QIAgen RNA clean-up kit (QIAgen, USA) as indicated by the manufacturer.

For qPCR analysis, 5ug of total RNA was converted into cDNA using Superscript III Reverse Transcriptase (Invitrogen, USA) in a cocktail containing 0.5mM dNTP mix, and 250ng of oligo (dT)_12-18_ (Invitrogen) in 1X First strand Buffer with 0.1M DTT (Invitrogen) and RNase OUT Recombinant RNase Inhibitor (Invitrogen). The cDNA was then added to a master mix of iTaq Universal SyBr Green Supermix (Bio-Rad), primers and sterile water and run on a T100 Thermal Cycler (25°C for 5 minutes, 55°C for 60 minutes, and 70°C for 15 minutes). The qPCR was run in triplicate on a 96-well plate using three biological replicates per sample on an Applied Biosystems 7500 Fast Real-Time PCR System machine. The primer sequences used were: SLK, 5′-CTTCAGGCGCTTTGAGCAGG-3′, and 5′-TTCTTGTTCCTCCTTCTTGCGGT-3′; E- Cadherin, 5′-CTTCCGAAAAGAAGGCTGTCC-3′ and 5′-CAGGTCTCCTCATGGCTTTGC-3′; Snai1, 5′-GTC AGCAAAAGCACGGTTG-3′ and 5′-CTTGTGTCT GCACGACCT-3′, Snail2 5′-GATGTGCCCTC AGGTTTGAT-3′ and 5′-GGCTGCTTCAAGGACA CATT-3′, Vimentin, 5′-CACATCGATCTGGACA TGCTGT-3′ and 5′-CGGAAAGTGGAATCCTTGCA-3′, Twist1, 5′-GGGACGCGGACATGGACC-3′ and 5′-CAC GCTGCCCTCGGACAA-3′, Twist2, 5′-GTCA TGAGGAGCCACAAGGT-3′ and 5′-ATGTCCGC CTCCCACTAGC-3′, Fibronectin, 5′-GCCCAGTGATT TCAGCAAAGG-3′ and 5′-ATGTGGACCCCTCCT GATAGT-3′.

### Subcellular fractionation

Subcellular fractionation was used to effectively separate the nuclear and cytoplasmic fractions in NMuMG cells. The cells were grown to approximately 75% confluency on 10cm plates and washed once in 1X PBS and once in 1X PBS supplemented with 1mM EDTA (Ethylenediaminetetraacetic acid). Cells were collected using a cell scraper, and centrifuged at 1300 x g for 5 minutes to pellet the cells. The cells were lysed in the cytoplasmic lysis buffer (300mM sucrose, 20mM HEPES (pH 7.4), 0.5% NP-40, 50mM NaCl, 3mM MgCl_2_, 1M NaF, 1M β- glycerophosphate, 1M DTT, 0.2M NaVO_3_, 0.1M PMSF, 1mg/mL leupeptin, 1mg/mL pepstatin, 1mg/mL aprotinin, 100μM benzamide, 1M Tris, 0.05% SDS, 1% Triton X-100, 1% Igepal CA-630, 50 mM Tris-HCl, pH 7.5, 150 mM NaCl, 2 mM EDTA, pH 8.0, and 12 mM Na-Deoxycholate) and then centrifuged at 1300g. The cytoplasmic fraction was collected and the pellet was lysed in nuclear lysis buffer (20mM HEPES (pH 7.4), 1% NP-40, 25 mM NaCl, 1.5mM MgCl_2_, 1M NaF, 1M β- glycerophosphate, 1M DTT, 0.2M NaVO_3_, 0.1M PMSF, 1mg/mL leupeptin, 1mg/mL pepstatin, 1mg/mL aprotinin, 100μM benzamide, 1M Tris, 0.05% SDS, 1% Triton X-100, 1% Igepal CA-630, 50 mM Tris-HCl, pH 7.5, 150 mM NaCl, 2 mM EDTA, pH 8.0, and 12 mM Na-Deoxycholate)) and then centrifuged at 1300g for 5 minutes. The pellet, containing the nuclear fraction was re-suspended in RIPA lysis buffer (1M NaF, 1M β- glycerophosphate, 1M DTT, 0.2M NaVO_3_, 0.1M PMSF, 1mg/mL leupeptin, 1mg/mL pepstatin, 1mg/mL aprotinin, 100μM benzamide, 1M Tris, 0.05% SDS, 1% Triton X-100, 1% Igepal CA-630, 50 mM Tris-HCl, pH 7.5, 150 mM NaCl, 2 mM EDTA, pH 8.0, and 12 mM Na-Deoxycholate). The suspension was cleared by centrifugation 20, 000g and the supernatant was used as the nuclear fraction.

## SUPPLEMENTARY MATERIALS FIGURES



## Abbreviations

bHLH: Basic helix-loop-helix
BMP: Bone morphogenetic protein
DAPI 4′: 6-diamidino-2-phenylindole
ECM: Extracellular matrix
EMT: Epithelial-to-mesenchymal transition
HGF: Hepatocyte growth factor
FA: Focal adhesion
FAK: Focal adhesion kinase
PVDF: Polyvinylidene fluoride
TβRI: Transforming growth factor-β receptor I
TβRII: Transforming growth factor-β receptor II
TGFβ: Transforming growth factor-β
TJ: Tight junction
ZEB: Zinc-finger E-box-binding homeobox
ZO-1: Zonula occludens-1
