# Supplementary material for: Transforming growth factor β-induced epithelial to mesenchymal transition requires the Ste20-like kinase SLK independently of its catalytic activity
Source: Oncotarget. 2017 Oct 19;8(58):98745–56. doi: 10.18632/oncotarget.21928 (PMC5716764; doi:10.18632/oncotarget.21928)
Supplement: Supplementary file 1 [file oncotarget-08-98745-s001.pdf]

Transforming growth factor β-induced epithelial to mesenchymal transition requires the Ste20-like kinase SLK independently of its catalytic activity

SUPPLEMENTARY MATERIALS

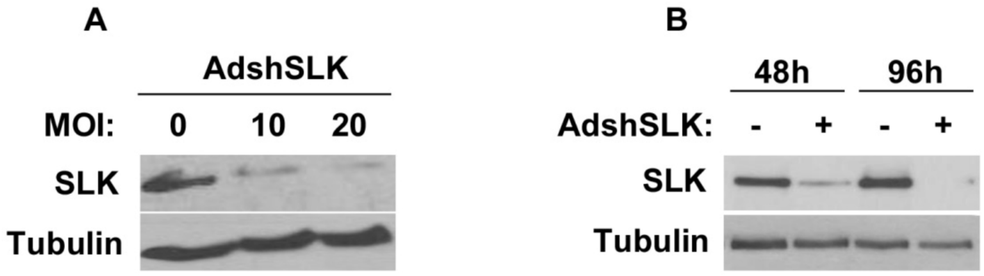

Supplementary Figure 1: Efficient knock down of SLK protein using Adenovirus. **(A)** NMuMG cells were infected at the indicated MOI and surveyed for SLK expression. Tubulin was used as a loading control. **(B)** NMuMG cells were infected at an MOI of 20 and assessed for SLK expression 48 and 96 hours following infection. Downregulation was observed for up to 9 days (not shown).

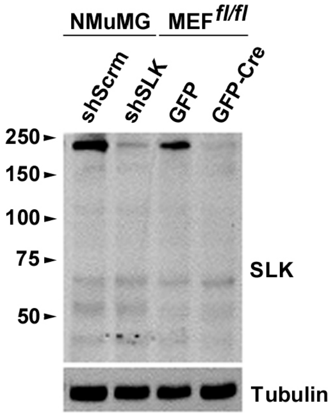

Supplementary Figure 2: AdshSLK knocks down all SLK reactive species in NMuMG. Full western blot showing that AdshSLK knocks down all SLK reactive species in NMuMG cells. The shSLK was directly compared to SLK knock-out fibroblasts (GFP-Cre) isolated from SLK conditional mice (MEF $^{fl/fl}$). The residual bands after knock out or knock down are indistinguishable, suggesting that all SLK isoforms have been depleted. Minor background bands are observed at ~70, 60 and 40 kDa.

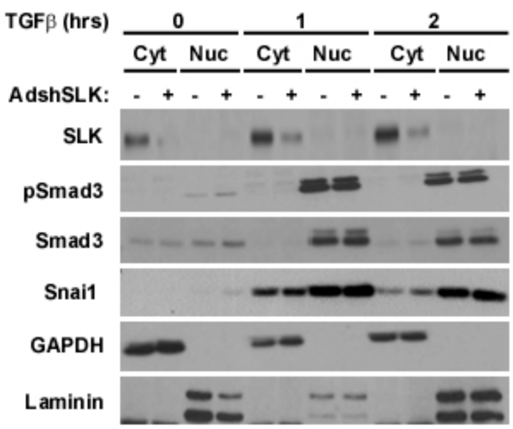

Supplementary Figure 3: SLK does not affect the Smad-dependent pathway of TGFβ-induced EMT. (A) NMuMG cells were first infected with AdshSCRM (-) or AdshSLK (+) for 48 hours prior to treatment with 2ng/mL of TGFβ1 for 1 or 2 hours. A subcellular fractionation was performed to effectively separate the cytoplasmic and nuclear fractions. The activation and translocation of Smad3 and Snail1 was analysed by Western blot. Cyt, cytoplasmic; Nuc, Nuclear. GAPDH and Lamin A/C were used as cytosolic and nuclear fraction markers, respectively.

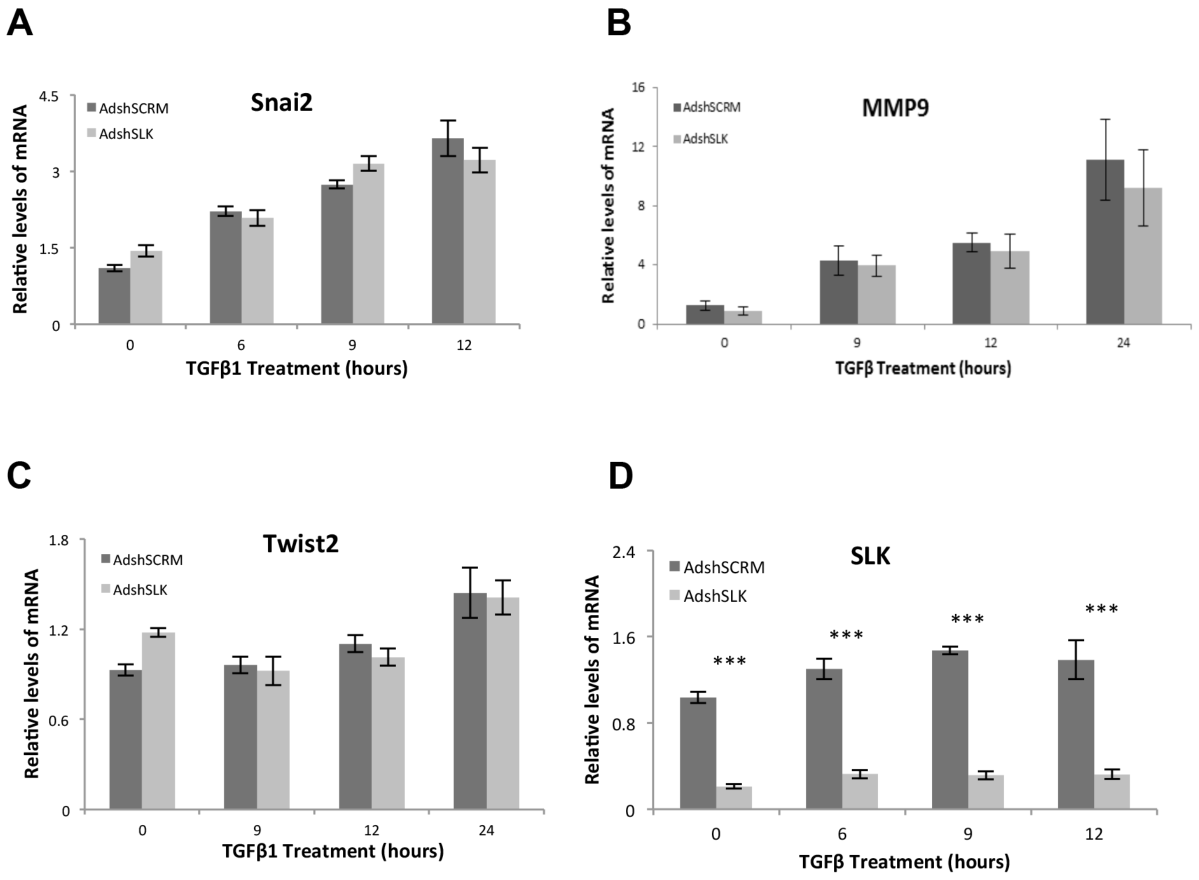

Supplementary Figure 4: SLK knock down does not affect the expression of Snail2, MMP9 or Twist2 following TGFβ stimulation. Total RNA was extracted following SLK knock down and TGFβ stimulation of NMuMG. Samples were assessed for the expression of Snail2, **(A)** MMP9, **(B)** Twist2 **(C)** or SLK **(D)** at the indicated time points post-stimulation. Q-PCR was normalized to GAPDH levels. Samples were evaluated in triplicate for n=3. *** $p<0.001$.

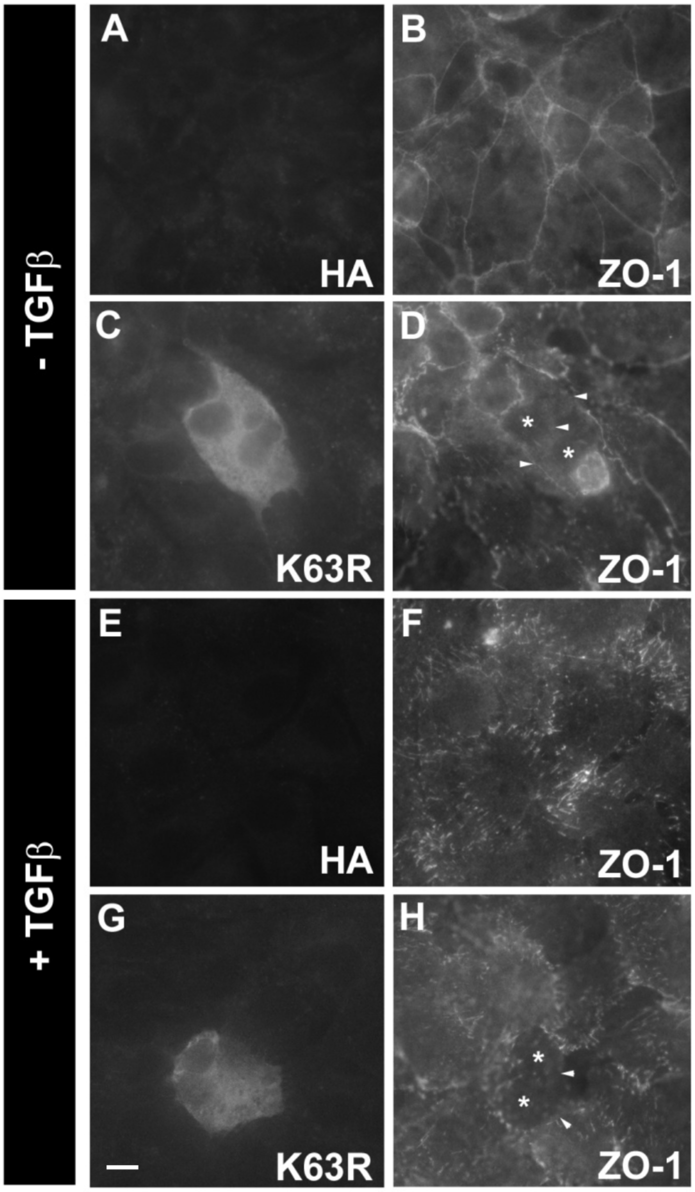

Supplementary Figure 5: Expression of kinase inactive SLK does not impair tight junction breakdown. NMuMG cells were transfected at low efficiency and serum starved 24h post-transfection. Cultures were then stimulated with TGFβ (2 ng/mL) for 48h prior to fixing and staining with anti-HA (panels **A**, **C**, **E** and **G**) and ZO-1 (panels **B**, **D**, **F** and **H**). Panels A and E show the HA vector control transfection. Panels C and G show a cluster of cells (asterisks) expressing the kinase inactive SLK (K63R). Tight junctions (arrowheads) were found to breakdown in K63R-expressing cells upon TGFβ stimulation. Scale bar=10μm
